# Multidimensional frailty and sleep quality in late adulthood: A UK biobank examination

**DOI:** 10.1111/jsr.14359

**Published:** 2024-09-23

**Authors:** Sarah P. Coundouris, Julie D. Henry, Lena K. L. Oestreich

**Affiliations:** ^1^ School of Psychology The University of Queensland Brisbane Queensland Australia; ^2^ Centre for Advanced Imaging and Australian Institute for Bioengineering and Nanotechnology The University of Queensland Brisbane Queensland Australia; ^3^ National Imaging Facility The University of Queensland Brisbane Queensland Australia

**Keywords:** cognitive frailty, longitudinal, physical frailty, psychological frailty, sleep quality, social frailty

## Abstract

Frailty and sleep disturbances are two major concerns in late adulthood, that not only profoundly threaten health and wellbeing at the individual level but place enormous demands on our healthcare systems. Given that both constructs represent dynamic states that are preventable and reversible, understanding the potential pathways to and effects of these variables on one another is critical in providing effective and tailored support. However, despite growing interest in the relationship between sleep and frailty, only one study to date has directly explored their potential bidirectionality. Accordingly, this study was designed to extend the current understanding by investigating the reciprocal relationship of frailty and sleep quality at the multidimensional level. Specifically, the bidirectionality of these relationships was considered separately for physical, psychological, cognitive, and social frailty. Four random‐intercept cross‐lagged panel models with three time points were conducted, using 3192 older adults (M_age_ = 60.21; 46.37% female at baseline) from the UK Biobank. The results revealed that while physical, psychological, and cognitive frailty were neither predictive of, nor predicted by, sleep quality, social frailty and sleep share a reciprocal relationship. These data therefore offer important preliminary evidence for the efficacy of early intervention and prevention strategies aimed at enhancing sleep quality to reduce social frailty, and vice versa.

## 1 INTRODUCTION

Frailty broadly refers to a complex, intermediate state of vulnerability to endogenous and exogenous stressors. While early conceptualisations of frailty focussed on physical components (e.g., weight loss, exhaustion; Fried et al., 2001), more contemporary conceptualisations acknowledge its multidimensional nature, and particularly how frailty also encompasses psychological, cognitive, and social components (De Vries et al., 2011; van Campen, 2011). Critically, although frailty increases one's vulnerability to adverse health and wellbeing outcomes – including disability, hospitalisation, and death – it is a dynamic condition that is both preventable and reversible (e.g., Kojima et al., 2018; Marcucci et al., 2019). With the proportion of older people in the global population growing at an unprecedented rate, and a longer life associated with increased risks of disease and dependency (WHO, 2022), identifying the factors that predict the onset and worsening of frailty in late adulthood is now critical.

Like frailty, sleep disturbances represent a major public health concern (Chattu et al., 2018). Sleep problems occur with increasing prevalence in late adulthood and can include (but are not limited to) reductions in sleep duration, efficiency, and general sleep quality, as well as increased sleep latency and wake after sleep onset (Miner & Kryger, 2020). Sleep disorders such as insomnia, are also frequently observed in older age and are associated with a heightened risk of developing physiological, psychological, and neurological disorders (de Almondes et al., 2016; Hertenstein et al., 2019; Javaheri & Redline, 2017).

Despite a growing interest in the relationship between sleep and frailty, to date only one study has longitudinally examined the potential bidirectionality of these two constructs using a multidimensional conceptualisation of frailty. Specifically, Nemoto et al. (2021) conducted a two‐year longitudinal study with a rural Japanese sample of community‐dwelling older adults, with data revealing equal reciprocal relationships after adjusting for common causes of sleep and frailty at baseline, suggesting that both frailty and insomnia are equivalent risk factors for the other. However, a key limitation of this study was that frailty was considered only as a single construct, rather than examining the potential unique relationships each separate dimension may share with sleep. Accordingly, the aim of the present study was to provide the first examination of the nature of the relationships between each frailty dimension and sleep. Specifically, the bidirectionality of these relationships was considered separately for physical, psychological, cognitive, and social frailty.

To date, research has predominantly focussed on the effects of sleep on frailty, with longitudinal studies indicating that poorer sleep quality is a significant predictor of increased physical, psychological, cognitive, and social frailty in older adults (e.g., Cheung et al., 2020; Cox et al., 2019; Ensrud et al., 2012; Jaussent et al., 2011; Lee et al., 2013; Nakakubo et al., 2019; Xu et al., 2024). In contrast, few longitudinal studies have examined how frailty impacts sleep quality (Smagula et al., 2016), although reviews focussed on these relationships suggest the possibility of a reinforcing cycle (e.g., Magee & Carmin, 2010; Vaz Fragoso et al., 2009).

As mentioned earlier, no singular study has explored the potentially distinct relationships that each individual frailty dimension may have with sleep. Even more surprising is that only five longitudinal studies, each focussing on a single frailty dimension (except for physical frailty), have statistically considered the bidirectionality of this relationship in late adulthood. Kim et al. (2009) revealed that insomnia was closely and reciprocally related to psychological frailty (depression) in a community‐dwelling sample of older adults. Yaffe et al. (2007) found some evidence for the bidirectionality of sleep and cognitive frailty, however, conclusions here were limited by a lack of sleep disturbance indicator at baseline, and a female only sample. Finally, three studies have used longitudinal designs to directly investigate the reciprocal association of specific components of social frailty and sleep, with some mixed findings noted. Cheng et al. (2018) identified reciprocal relationships between social network strength and sleep disturbance in community‐dwelling older adults across a 6 year period. Further evidence for bidirectionality was observed by Griffin et al. (2020), in which small reciprocal associations were found between loneliness and sleep disturbance in an older adult sample across 8 years. However, most recently Xu et al. (2021) found that sleep quality predicts perceived social support and not vice versa, but acknowledged the lack of reciprocal causality may have been an artefact of the relatively short timeframe used, with participants examined three times within 1 year.

### 1.1 Study overview

This study provides an important replication and extension of longitudinal studies that have used cross‐lagged panel modelling to provide insight into the directionality of frailty and sleep (Cheng et al., 2018; Griffin et al., 2020; Nemoto et al., 2021; Xu et al., 2021). Specifically, the current study was not only the first to explore the separate reciprocal relationships between each frailty dimension (i.e., physical, psychological, cognitive, and social frailty) and sleep, but to also use broader operationalisations of each of these constructs (i.e., multiple indicators were used to operationalise each frailty dimension and sleep). This study therefore provides the most comprehensive examination of the bidirectionality of sleep and frailty to date. In line with previous findings, it was pre‐registered on the Open Science Framework (OSF) that each frailty dimension would be bidirectionally related with sleep quality.

## 2 METHOD

The present study's design, aims and hypotheses were pre‐registered on OSF (https://osf.io/xj7ge/).

### 2.1 Participants

The present study used three waves of data (Baseline/Instance 0, 2006–2010; Instance 2, 2014+; Instance 3, 2019+), taken from the UK Biobank Data from Instance 1 (2012–2013) was omitted from the present study due to a reported error with hand grip data, and to allow for the inclusion of numeric memory. The UK Biobank is a large community‐based cohort, comprising individuals aged 37 years and above across the United Kingdom. Due to the focus in late adulthood, only participants aged 55 and above at baseline (Instance 0) were eligible to contribute. Additionally, due to the smaller number of individuals contributing to Instance 3, only individuals contributing data at Baseline and this timepoint were included. The final sample comprised 3192 individuals aged 55–70 (M = 60.21, SD = 3.72) at baseline (46.37% female). Further demographic information can be found in Supplementary 1.

### 2.2 Measures

#### 2.2.1 Frailty

While the literature commonly classifies individuals into one of three frailty stages – not frail, pre‐frail, and frail – given that frailty develops as a continuum, the present study considered frailty as a continuous variable. Additionally, as mentioned previously, four separate frailty scores (one for each dimension; physical, psychological, cognitive, and social) were created to provide a more nuanced and comprehensive understanding of any relationships between sleep and frailty. Following Rockwood and Mitnitski's (2007) approach whereby frailty represents a cumulative count of deficits, for each question, responses were assigned between 0 and 1 according to severity (0 meaning a deficit is absent, and 1 meaning the deficit is at its most severe). This allowed for different response formats (dichotomous, Likert‐scale) to be combined equitably. A detailed account of how each criterion was coded/calculated was pre‐registered and is reported in Supplementary 2.

##### Physical frailty

The physical frailty component was operationalised in accordance with the subdomains captured by two commonly used criteria: The Fried Phenotype (Fried et al., 2001) and the physical component of the Tilburg Frailty Indicator (TFI; Gobbens et al., 2010). Specifically, the physical frailty score comprised: low handgrip strength, exhaustion, low physical activity, perceived health, walking, and poor hearing and vision [While both unintentional weight loss and balance are also considered within these two criteria, as accurate indicators were not available within the UK Biobank dataset, no questions were included in relation to these]. Total scores ranged from 0 to 11, with higher scores representing greater physical frailty.

##### Psychological frailty

The psychological frailty component was primarily operationalised in accordance with the TFI (Gobbens et al., 2010) which defines psychological frailty as self‐reported feelings of anxiety, depression, and declines in coping and memory. The only deviation here was that, we have chosen to distinguish memory decline into its own frailty category – cognitive frailty. This decision was made to allow for a more in‐depth measurement of cognitive frailty that considers domains other than just memory (see next section for details). Total scores ranged from zero to seven, with higher scores representing greater psychological frailty.

##### Cognitive frailty

In 2013, the International Academy on Nutrition and Aging and the International Association of Gerontology and Geriatrics provided the first formal definition of cognitive frailty: the co‐occurrence or incidence of physical frailty and cognitive impairment without a clinical diagnosis of dementia (Kelaiditi et al., 2013). In line with other studies which have separately evaluated all four dimensions of frailty (e.g., van Oostrom et al., 2017), here, we considered only the *distinct cognitive* features comprising cognitive frailty. Unfortunately, as a robust and widely accepted operational criteria is yet to be established, such features have been inconsistently captured across studies. This includes self‐reported cognitive screening questionnaires, neuropsychological batteries, generalised cognitive assessments, and/or the presence of delirium/clouding of consciousness (Azzopardi et al., 2018).

Critically, in the selection of objective indicators of cognitive frailty the literature highlights the value of (1) specific cognitive assessments over generalised screening measures; and (2) the inclusion of domains known to be affected by ageing (Azzopardi et al., 2018). Consequently, although the present study was limited to the selection of cognitive measures available within the UK Biobank dataset, we not only ensured that multiple cognitive domains were included, but also that each of these domains have been shown to decline with age.

Specifically, cognitive frailty was assessed with a cognitive test battery. Measures of reaction time, fluid intelligence/reasoning, and visual and numeric memory were included, with each of these shown to generally decline in older age. For each measure, total scores were converted into *z*‐scores before averaging all measures together to create a composite cognitive frailty score. In contrast to the other domains, higher scores were representative of lower frailty.

##### Social frailty

Social frailty was operationalised in line with Bessa et al.'s (2018) classification of the different social frailty representations. The benefit of this approach is that this classification captures both the perception of inadequate social needs fulfilment, as well as objective insufficiency. This is important given that a recent 6 year longitudinal study suggested objective and subjective representations of social frailty follow distinct pathways in affecting sleep quality in older adults (Yu et al., 2018). Questions relating to objective (living alone, social network, social activities) and subjective (social support, loneliness) social frailty were included. Total scores ranged from zero to seven, with higher scores representing greater social frailty.

#### 2.2.2 Subjective sleep quality

To optimise the subjective sleep quality assessment in the UK Biobank, the present study followed Gao et al.'s (2022) six‐item adaption of the UK Biobank algorithm. This version also considers the ease of getting up in the morning, alongside chronotype, duration, insomnia, snoring, and excessive daytime sleepiness to create a healthy sleep score. As per the pre‐registration, low‐risk factors receive a score of 1 and were defined as: getting up easily in the morning ("fairly easy" or "very easy"), an early chronotype ("morning" or "morning than evening"), 7–8 h daily sleep duration, never or rarely insomnia symptoms, no self‐reported snoring, and no frequent daytime sleepiness ("never/rarely" or "sometimes"). All component scores were summed to create a healthy sleep score ranging from zero to six, with higher scores indicating greater sleep quality. The biobank field coding for these variables is available within Supplementary 3.

### 2.3 Data analyses

Before testing the random intercept cross‐lagged panel models (RI‐CLPM; Hamaker et al., 2015), descriptive statistics and bivariate correlations between the main variables and potential time‐invariant (age, sex) and time‐variant covariates (employment status, alcohol consumption, and smoking status) were calculated.

Separate RI‐CLPMs were performed to infer the temporal relationship of sleep with each frailty dimension across three waves (a conceptual overview including Figure is provided in Supplementary 6). The RI‐CLPM was chosen over the traditional cross‐lagged method as the latter assumes that relationships are the same for all individuals and that there are no individual differences in the starting levels (intercepts). Thus, the RI‐CLPM is more appropriate as the model estimates the random intercepts for each individual, thereby decomposing the observed variance into stable, between‐unit differences versus temporal, within‐unit dynamics. Further, RI‐CLPM tends to fit empirical data (much) better than the traditional panel model (Hamaker et al., 2015).

In RI‐CLPMs, *random intercepts* capture stable, individual‐specific differences across time, accounting for variability between individuals in their average levels of the variables; *lagged* (*autoregressive*) *effects* reflect the influence of a variable on itself across subsequent time points, indicating stability over time; and *cross‐lagged effects* show how one variable at an earlier time point predicts another variable at a later time point, highlighting the temporal relationships between variables.

Analyses were completed using the Lavaan package (Rosseel, 2012) in R Studio, following the suggested procedure by Hamaker et al. (2015). Specifically, the unconstrained model was compared against a model constrained to be equal over time (fixed auto‐regressive and cross‐lagged relations, time‐invariant (residual) (co‐)variances in the within‐person part, and constrained grand means). As constraints are placed on the bound of the parameter space, a chi‐bar‐square test (*ChiBarSq.DiffTest* package; Kuiper, 2021), rather than the traditional chi‐square difference test was used to compare these two models (Mulder & Hamaker, 2021). Additionally, in line with Mulder and Hamaker (2021), Bayesian information criterions (BIC) were also used as measures of model fit, where the model with the lower BIC was preferred. Finally, root mean square error of approximation (RMSEA), comparative fit index (CFI), and standardised root mean square residual (SRMR) were also considered in the evaluation of model fit, whereby models where RMSEA and SRMR <0.05, and CFI >0.95, were considered to have an acceptable fit in accordance with recommended cut offs (Hooper et al., 2007).

Socio‐demographic variables (age, sex, employment status [worker, non‐worker]), alcohol consumption (almost every day, or other), smoking status (current smoker or other) were included as covariates within the analyses. The contributions of these covariates on the observed outcome variables were estimated at each wave. Finally, missing data were handled using the full information maximum likelihood method, allowing for more robust and accurate parameter estimation even when data are missing at random.

## 3 RESULTS

### 3.1 Model fits

The chi‐bar‐square test revealed non‐significant differences between the basic and constrained models for each frailty dimension and sleep RI‐CLPM. Except for the physical frailty and sleep RI‐CLPM, all models estimated with constraints fitted the data best; psychological frailty BICs = 51,300 (basic) and 51,244 (constrained), cognitive frailty BICs = 32,036 (basic) and 31,986 (constrained), social frailty BICs = 41,523 (basic) and 41,493 (constrained). An evaluation of other model fit criteria revealed an acceptable fit of these constrained models, RMSEAs = 0.03, SRMRs = 0.02, CFIs = 0.99.

For physical frailty, although the basic model emerged as the better fit according to the BIC, the goodness of fit indices still signified good fit for the constrained model, RMSEA = 0.03, SRMR = 0.03, CFI = 0.97. Given that (1) the constrained model was still an acceptable fit, and (2) the pattern of results for the key variables did not differ between the models, the constrained model is reported below for all four models for consistency. As adding equality constraints did not deteriorate model fit, we can conclude that the effects from Time 1 to Time 2 are similar to the effects from Time 2 to Time 3. Statistics related to all model covariates are provided in Supplementary 5.

### 3.2 Lagged (autoregressive) effects

As may be seen in Table 1, apart from cognitive frailty, the RI‐CLPMs revealed past frailty to be predictive of current frailty. Specifically, greater physical, psychological and/or social frailty at one time point was predictive of greater levels of the respective frailty dimension at the next time point, demonstrating stability over time. Interestingly, the meaningfulness of these lagged effects varied considerably, with this relationship being strongest for physical frailty (large effect size), followed by social frailty (moderate effect size) and psychological frailty (small effect size). Similarly, a significant small autoregressive effect of sleep was found, such that poorer sleep quality at one time point was predictive of poorer sleep quality at the next.

**TABLE 1:** Summary of the main parameters for each RI‐CLPM model – Frailty and sleep across three waves of data.

	Physical frailty and sleep			Psychological frailty and sleep			Cognitive frailty and sleep			Social frailty and sleep		
	β	SE	*p*	β	SE	*p*	β	SE	*p*	β	SE	*p*
Lagged (autoregressive) effects												
Frailty T1 ➔ Frailty T2	**0.50**	**0.05**	**<0.001**	**0.12**	**0.02**	**<0.001**	0.12	0.08	0.091	**0.30**	**0.06**	**<0.001**
Frailty T2 ➔ Frailty T3	**0.58**	**0.05**	**<0.001**	**0.09**	**0.02**	**<0.001**	0.13	0.08	0.091	**0.31**	**0.06**	**<0.001**
Sleep T1 ➔ Sleep T2	**0.14**	**0.03**	**<0.001**	**0.14**	**0.03**	**<0.001**	**0.14**	**0.03**	**<0.001**	**0.14**	**0.03**	**< 0.001**
Sleep T2 ➔ Sleep T1	**0.13**	**0.03**	**<0.001**	**0.13**	**0.03**	**<0.001**	**0.13**	**0.03**	**<0.001**	**0.13**	**0.03**	**<0.001**
Cross‐lagged effects												
Frailty T1 ➔ Sleep T2	−0.01	0.03	0.797	−0.004	0.02	0.891	0.06	0.10	0.142	**−0.10**	**0.05**	**0.008**
Frailty T2 ➔ Sleep T3	−0.01	0.03	0.797	−0.003	0.02	0.891	0.07	0.10	0.142	**−0.11**	**0.05**	**0.008**
Sleep T1 ➔ Frailty T2	0.03	0.05	0.436	0.004	0.02	0.878	0.07	0.02	0.066	**−0.07**	**0.02**	**0.022**
Sleep T2 ➔ Frailty T3	0.02	0.05	0.436	0.003	0.02	0.878	0.06	0.02	0.066	**−0.06**	**0.02**	**0.022**
Random intercepts												
Frailty	**2.67**	**0.05**	**<0.001**	**1.30**	**0.02**	**<0.001**	**0.22**	**0.02**	**<0.001**	**2.05**	**0.02**	**<0.001**
Sleep	**3.96**	**0.02**	**< 0.001**	**3.96**	**0.02**	**<0.001**	**3.96**	**0.02**	**<0.001**	**3.96**	**0.02**	**<0.001**
Covariances												
Frailty (kappa) ➔ Sleep (omega)	**−0.46**	**0.05**	**<0.001**	**−0.24**	**0.02**	**<0.001**	−0.08	0.01	0.120	**−0.14**	**0.02**	**<0.001**
Frailty T1 ➔ Sleep T1	0.03	0.05	0.579	**0.01**	**0.02**	**<0.001**	0.10	0.02	0.114	−0.03	0.02	0.470
Frailty T2 ➔ Sleep T2	−0.04	0.03	0.287	**−0.07**	**0.01**	**0.006**	0.05	0.01	0.233	**−0.12**	**0.01**	**<0.001**
Frailty T3 ➔ Sleep T3	−0.04	0.03	0.287	**−0.07**	**0.01**	**0.006**	0.05	0.01	0.233	**−0.12**	**0.01**	**<0.001**

*Note*: *N* = 3192. Significant paths in bold.

Abbreviations: β, standardised coefficient; RI‐CLPM = random‐intercept cross lagged panel model; SE, standard error.

### 3.3 Cross‐lagged effects

**FIGURE 1:**
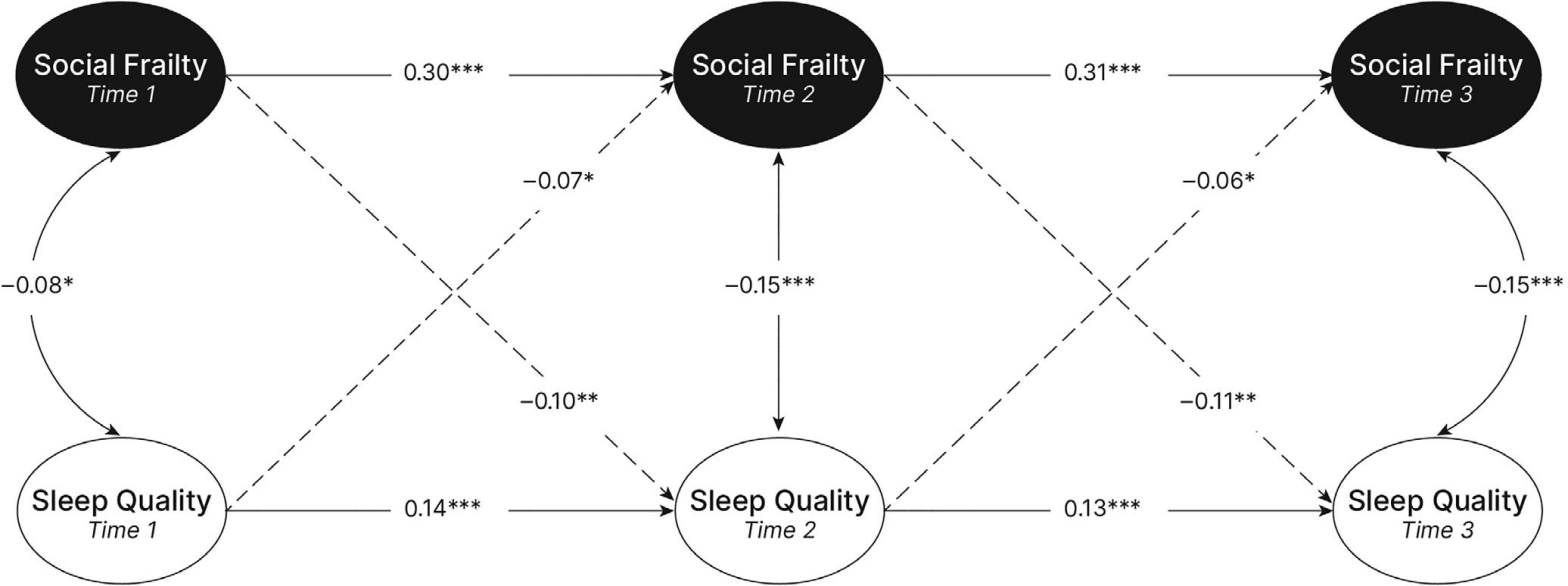
Simplified representation of the social frailty and sleep random intercept cross‐lagged panel model (RI‐CLPM) across three time points. Diagonal dashed arrows represent the cross‐lagged effects, while horizontal arrows represent the lagged (autoregressive) effects; the standardised coefficients (*β*) are displayed for all these effects. Vertical/curved arrows represent correlations. ***p < 0.001, **p < 0.01, *p < 0.05.

As may be seen in Table 1, neither physical, cognitive, nor social frailty were predictive of nor predicted by sleep quality; that is no significant cross‐lagged effects were found within these three RI‐CLPMs. Alternatively, evidence for bidirectionality of the relationship between social frailty and sleep from T1–T2 and T2–T3 were found and is displayed visually in Figure 1. Specifically, comparably sized (albeit small) inverse effects were found, suggesting that greater social frailty (e.g., greater feelings of loneliness, lower social network size, and social support etc.) was both predictive of, and predicted by, lower sleep quality.

## 4 DISCUSSION

The present study provides the most comprehensive longitudinal examination of the bidirectionality between sleep and frailty available to date, by being the first to (1) consider frailty at the multidimensional level (i.e., physical, psychological, cognitive, and social frailty examined separately), and (2) use broader operationalisations of each of these constructs (i.e., multiple indicators to operationalise each frailty dimension and sleep). Contrary to expectations, neither physical, psychological nor cognitive frailty were found to be predictive of, or predicted by, sleep quality. However, in line with previous literature, small reciprocal associations between social frailty and sleep were revealed (Cheng et al., 2018; Griffin et al., 2020; Xu et al., 2021).

One interpretation of the study's findings is that, of the four frailty dimensions, social frailty is the only one significantly impacting, and being impacted by sleep. In other words, it may be that Nemoto et al. (2021)'s findings of equal reciprocal relationships between frailty and sleep were being primarily driven by social frailty. The emergence of a bidirectional relationship between sleep quality and social frailty, points to the need to consider both of these variables in the tailoring of effective care and support interventions for older adults. While the magnitude here was small (in line with previous literature; Cheng et al., 2018; Griffin et al., 2020; Xu et al., 2021), the fact that these relationships emerged with a sample predominantly in the early stages of older adulthood, signify the robustness and sensitivity of these relationships, and particularly the strategic value of early interventions.

In regard to potential mechanisms, the broader literature suggests associations with social frailty and heightened vulnerability and vigilance towards potential environmental threats, with this implicit feeling of being unsafe interfering with sleep efficiency and quality (Hawkley & Cacioppo, 2010; Segrin & Burke, 2015). This makes sense from an evolutionary perspective, whereby a safe environment has been needed for protection from danger while sleeping, and this need is typically met by living with/being connected to others. Poorer sleep has also been proposed to instigate a propagating, self‐reinforcing cycle of social separation and withdrawal (Simon & Walker, 2018). Given that sleep is a resource of recovery, continuous poor sleep quality may leave older adults with inadequate resources to initiate and maintain social relationships. Overall, these findings highlight the complex interplay between social frailty and sleep quality, suggesting that impaired sleep may exacerbate social withdrawal, creating a reinforcing cycle that undermines social engagement in older adults.

However, given that previous literature, albeit limited, supports the reciprocal associations between sleep and the other frailty dimensions (e.g., Cox et al., 2019; Kim et al., 2009; Yaffe et al., 2007), the unexpected results may be attributed to methodological differences. For example, our older adult sample were predominantly young (baseline minimum age = 55, M_age_ = 60.21, SD = 3.72), whereas in these other studies the most frequent minimum age inclusion was 65. It therefore may be that the detrimental effects of the other frailty dimensions and sleep only emerge at a later stage of old age (e.g., 70s and above); and indeed, supporting this premise, our cohort also generally reported low frailty. An important future study is therefore to repeat the current study with the UK Biobank cohort in 10 years' time.

Another possible explanation for the null findings is the way in which the variables were operationalised. While the UK Biobank afforded excellent power to longitudinally examine the bidirectionality of frailty and sleep across three time points, a limitation is that we were restricted in how constructs were measured. Although broad operationalisations were used (i.e., multiple indicators used to operationalise each frailty dimension and sleep) based off formal definitions of each frailty dimension, some considerations should be noted.

Firstly, the current dataset did not allow for the inclusion of unintentional weight loss or balance, and it may be that one or both these factors are particularly important in the relationship between physical frailty with sleep. Indeed, while the literature on unintentional weight loss (rather than gain) is extremely limited, prior research has indicated that older adults reporting sleep disturbances are at a higher risk of falling and having poorer functional balance (e.g., Takada et al., 2018). Secondly, in terms of psychological frailty, while questions were included that related to each of the three distinct elements (anxiety, depression, and maladaptive coping), most of these questions failed to capture severity (i.e., were dichotomous yes/no answer format rather than the extent to which they were feeling a certain way). Future work should therefore endeavour to explore this question using more sensitive answer formats and/or formal diagnoses of psychological illness. Thirdly, with respect to cognitive frailty, while a noted strength of this study was that we ensured multiple cognitive domains were included, and that one's ability in each of these domains typically declines with age, given the large number of questionnaires/tasks administered to the UK Biobank cohort, only brief assessments of these cognitive capacities are conducted to minimise fatigue. Indeed, the cross‐lagged effect of sleep predicting cognitive frailty was trending towards significance so there is a possibility that with a more in‐depth battery of cognitive measures, this relationship may emerge; again, this is something future research should consider examining.

Finally, while a strength of this study was the inclusion of multiple indicators of sleep quality, each of these were *subjective* (i.e., self‐report) in nature. Sleep quality can also be assessed using objective measures such as wrist‐worn actigraphy and polysomnography. Investigation of the present study's models using objective indicator of sleep represents an important avenue for future research, given that broader literature in late adulthood has demonstrated that subjective and objective sleep measures capture unique aspects of sleep (e.g., Hughes et al., 2018). Therefore, to gauge the full effect of frailty on sleep and vice versa, and particularly whether different patterns of results emerge across the frailty dimensions, objective indicators of sleep are required. A study that allows for an examination of both objective and subjective indicators concurrently would be ideal, to understand whether it is one's perception of their sleep quality, or actual objective physiological estimates of sleep quality that are most influential to, and influenced by, frailty.

To conclude, sleep quality and frailty are two major concerns that cause significant health problems in late adulthood and present a public burden. Given that both frailty and sleep quality represent dynamic states that are preventable and reversible, understanding the potential pathways to and effects of these constructs on one another holds considerable worth in improving the health and wellbeing trajectories of older adults. Through mapping how change in one variable predicts the subsequent change in another across three time points, the RI‐CLPMs were able to provide unique insights into the directionality of sleep and frailty at the multidimensional level. The present study provided initial support for the efficacy of integrated prevention and early intervention strategies that focus on improving sleep quality and social frailty simultaneously. Further work is now needed to understand the mechanisms behind these relationships, including other alterable influential factors.

## FUNDING INFORMATION

This work was supported by an Australian Research Council Linkage Grant (LP190100761); and L.K.L Oestreich was supported by a National Health and Medical Research Council Investigator Grant (20077181), and a start‐up package from the Australian Institute for Bioengineering and Nanotechnology at the University of Queensland.

## CONFLICT OF INTEREST STATEMENT

We have no known conflict of interest to disclose.

## Supporting information


**DATA S1** Supporting information.

## Data Availability

The data that support the findings of this study are available from The UK Biobank. Restrictions apply to the availability of these data, which were used under license for this study. Data are available from https://www.ukbiobank.ac.uk/enable-your-research with the permission of The UK Biobank.
