# Supplementary material for: Multidimensional frailty and sleep quality in late adulthood: A UK biobank examination
Source: J Sleep Res. 2024 Sep 23;34(2):e14359. doi: 10.1111/jsr.14359 (PMC11911041; doi:10.1111/jsr.14359)
Supplement: Supplementary file 1 — DATA S1 Supporting information. [file JSR-34-e14359-s001.docx]

**Supplementary Table 1**

*Participant Demographic Information at Each Wave*

| **Demographic Characteristic** | *Baseline*  *n* (%) | *Time 1*  *n* (%) | *Time 2*  *n* (%) |
| --- | --- | --- | --- |
| Sex |  |  |  |
| *Male* | 1480 (46.37) | - | - |
| *Female* | 1712 (53.63) | - | - |
| Ethnicity |  |  |  |
| *White* | 3080 (96.49) | - | - |
| *Asian* | 17 (0.53) | - | - |
| *Black* | 8 (0.25) | - | - |
| *Mixed* | 4 (0.13) | - | - |
| *Don’t know/Prefer not to say* | 10 (0.31) | - | - |
| *No Response* | 73 (2.29) | - | - |
| Household Income |  |  |  |
| *< £18,000* | 434 (13.60) | 397 (12.44) | 370 (11.59) |
| *£18,000 - £30,999* | 782 (24.50) | 971 (30.42) | 957 (29.98) |
| *£31,000 - £51,999* | 872 (27.32) | 906 (28.38) | 916 (28.70) |
| *£52,000 - £100,000* | 644 (20.18) | 478 (14.97) | 517 (16.20) |
| *> £100,000* | 160 (5.01) | 101 (3.16) | 89 (2.79) |
| *Don’t know/Prefer not to say* | 264 (8.27) | 306 (9.59) | 333 (10.43) |
| *No Response* | 36 (1.13) | 33 (1.03) | 10 (0.31) |
| Employment Status |  |  |  |
| *Currently Employed* | 1707 (53.48) | 392 (12.28) | 241 (7.55) |
| *Other* | 1464 (45.86) | 2765 (86.82) | 2947 (92.32) |
| *No Response* | 21 (0.66) | 35 (1.10) | 4 (0.13) |
| Alcohol Consumption |  |  |  |
| *Daily/Almost Daily* | 826 (25.88) | 597 (18.70) | 600 (18.80) |
| *Other* | 2364 (74.06) | 2567 (80.42) | 2590 (81.14) |
| *No Response* | 2 (0.06) | 28 (0.88) | 2 (0.06) |
| Smoking |  |  |  |
| *Current smoker* | 152 (4.76) | 56 (1.75) | 56 (1.75) |
| *Other* | 3038 (95.18) | 2986 (93.55) | 2986 (93.55) |
| *No Response* | 2 (0.06) | 150 (4.70) | 150 (4.70) |

**Supplementary 2 - Frailty Categorisation and Coding**

*Physical Frailty*

Low handgrip strength, exhaustion, low physical activity, perceived health, walking, and poor hearing and vision

| **Subdomain** | **Question** | **Category/Field** | **Coding** |
| --- | --- | --- | --- |
| Low Handgrip Strength | Handgrip – left/right used depending on handedness | Handgrip 100019/46 &47  Handedness  100033/1707  BMI 100010/21001 | Cut-off points for handgrip strength in kg stratified by sex and BMI based on the Fried Phenotype (Fried et al., 2001).  Males received a one if hand grip strength ≤29.0 kg for BMI ≤24.0, ≤30.0 kg for BMI 24.1-26.0, ≤30 kg for BMI 26.1-28.0, and ≤32.0 kg for BMI >28.0.  Females received a one if hand grip strength ≤17.0 kg for BMI ≤23.0, ≤17.3 kg for BMI 23.1-26.0, ≤18.0 kg for BMI 26.1-29.0, and ≤21.0 kg for BMI >29.0 |
| Exhaustion | “Over the past two weeks, how often have you felt tired or had little energy?” | 100060/2080 | Not at all (0), several days (0.33), more than half (0.67), nearly every day (1) |
| Low Physical Activity | International Physical Activity Questionnaire (IPAQ Research Committee, 2005) low activity group assignment | 100054/22032 | Low (1), moderate (0), high (0) |
| Perceived Health | “In general how would you rate your overall health?” | 100042/2178 | Excellent (0), good (0. 33) fair (0.67) and poor (1) |
| Walking | 1. “Do you get short of breath walking with people of your own age on level ground?”  2. “Do you get a pain in either leg on walking?”  3. “How would you describe your usual walking pace?” | 1  100037/4717  2  100038/4728  3  100054/924 | 1 & 2: Yes (1), No (0)  3: Not collected for people who indicated that they are unable to walk, thus NA (1), slow pace (1), steady average pace (0), brisk pace (0), None of the above (1) |
| Poor Hearing | 1. “Do you have any difficulty with your hearing?”  2. “Do you find it difficult to follow a conversation if there is background noise (such as TV, radio, children playing)” | 100043/2247 & 2257 | 1: I am completely deaf (1), Yes (1), No (0)  2: If listed “I am completely deaf” in above (1), otherwise Yes (1), No (0) |
| Poor Vision | 1. “Do you wear glasses or contact lenses to correct your vision?”  2. “Has a doctor told you that you have any of the following problems with your eyes? [diabetes related eye disease, glaucoma, injury/trauma resulting in loss of vision, cataract, macular degeneration, other]” | 100041/2207 & 6148 | 1: Yes (1), No (0)  2: Allow multiple answers, however we only coded the first mention of disease (as interested in if have, not how many or what). Any disease, including “other” (1), None of the above (0) |

*Note.* Prefer not to answer and do not know recoded to 0 (i.e., no frailty).

*Psychological Frailty*

Depressive symptoms, anxiety symptoms and coping with problems

| **Subdomain** | **Question** | **Category/Field** | **Coding** |
| --- | --- | --- | --- |
| Depressive Symptoms | 1. “Do you ever feel 'just miserable' for no reason?”  2. “Over the past two weeks, how often have you felt down, depressed or hopeless?”  3. “Over the past two weeks, how often have you had little interest or pleasure in doing things?” | 100060/1930, 2050 & 2060 | 1: Yes (1), No (0)  2 & 3: Not at all (0), several days (0.33), more than half (0.67), nearly every day (1) |
| Anxiety Symptoms | 1. “Do you suffer from 'nerves'?”  2. “Over the past two weeks, how often have you felt tense, fidgety or restless?” | 100060/2010 & 2070 | 1: Yes (1), No (0)  2: Not at all (0), several days (0.33), more than half (0.67), nearly every day (1) |
| Coping with Problems | 1. “Are you a worrier?”  2. “Would you call yourself tense or 'highly strung'” | 100060/1980 & 1990 | 1 & 2: Yes (1), No (0) |

*Note.* Prefer not to answer and do not know recoded to 0 (i.e., no frailty).

*Cognitive Frailty*

Measures of reaction time, fluid intelligence/reasoning, and visual and numeric memory

| **Subdomain** | **Question** | **Category/Field** | **Coding** |
| --- | --- | --- | --- |
| Reaction Time | Timed test of symbol matching | 100032/20023 | Mean response time in milliseconds across trials which contained matching pairs |
| Fluid Intelligence/Reasoning | Thirteen verbal and numerical reasoning questions (e.g., addition, word interpolation, square sequence recognition) and included a two-minute limit | 100027/20016 | Fluid intelligence score (0-13) |
| Visual Memory | A pairs matching task (participants memorizing the position of six card pairs and then being asked to match them from memory with as few errors as possible) | 100030/399 | Number of incorrect matches^a^ |
| Numeric Memory | Numerical string recall (become sequentially longer) | 100029/4282 | Maximum digits correctly remembered |

*Note.* Prefer not to answer and do not know recoded to 0 (i.e., no frailty). Due to the longitudinal nature of the present study, cognitive assessments were only considered for inclusion if they had been assessed on three instances (0, 2, 3). Additionally, the prospective memory task was omitted due to the dichotomous nature of the task (i.e., did not capture differing degrees of prospective memory functioning).

^a^ Deviation from pre-registration where stated number of correct matches. Incorrect matches more accurate gauge of memory. This score was inverse scored to be in the same direction as other measures.

*Social Frailty*

Living alone, social network, social support, loneliness, and social activities

| **Subdomain** | **Question** | **Category/Field** | **Coding** |
| --- | --- | --- | --- |
| Living Alone | “Including yourself, how many people are living together in your household?” | 100066/709 | 1 (1), any other number (0) |
| Social Network | “How often do you visit friends or family or have them visit you?” | 100061/1031 | Almost daily (0), 2-4 times a week (0.20), about once a week (0.40), about once a month (0.60), once every few months (0.80), never or almost never (1). If participants selected “no friends/family outside the household” and reported living alone (1). otherwise (.20) |
| Social Support | 1. “How often are you able to confide in someone close to you?”  2. “In general how satisfied are you with your family relationships”  3. “In general how satisfied are you with your friendships” | 1  100061/2110  2 & 3  100060/4559 & 4570 | 1: Almost daily (0), 2-4 times a week (0.20), about once a week (0.40), about once a month (0.60), once every few months (0.80), never or almost never (1)  2 & 3: Extremely happy (0), very happy (0.20), moderately happy (0.40), moderately unhappy (0.60), very unhappy (0.80), extremely unhappy (1) |
| Loneliness | “Do you often feel lonely?” | 100060/2020 | Yes (1), No (0) |
| Social Activities | “Which of the following do you attend once a week or more often? [sports club/gym, pub/social club, religious group, adult education class, other]” | 100061/6160 | None of the social activities listed (1), one social activity (0.80), two social activities (0.60), three social activities (0.40), four social activities (0.20), five social activities (0)^a^ |

*Note.* Prefer not to answer and do not know recoded to 0 (i.e., no frailty).

^a^ This deviates from pre-registration which was incorrectly coded (i.e., higher score did not reflect greater social frailty

**Supplementary Table 3**

*Subjective Sleep Quality Categorisation*

| **Subdomain** | **Question** | **Category/Field** | **Coding** |
| --- | --- | --- | --- |
| Ease of Getting Up | “On an average day, how easy do you find getting up in the morning?” | 100057/1170 |  |
| Chronotype | “Do you consider yourself to be? Definitely a morning, more a morning than evening, more an evening than morning, definitely an evening” | 100057/1180 |  |
| Sleep Duration | “About how many hours sleep do you get in every 24 hours? (please include naps)” | 100057/1160 |  |
| Insomnia | “Do you have trouble falling asleep at night or do you wake up in the middle of the night?” | 100057/1200 |  |
| Snoring | “Does your partner or a close relative or friend complain about your snoring?” | 100057/1210 |  |
| Excessive Daytime Sleepiness | “How likely are you to doze off or fall asleep during the daytime when you don't mean to? (e.g. when working, reading or driving)” | 100057/1220 |  |

*Note.* Prefer not to answer and do not know recoded to 0.

**Supplementary Table 4**

*Bivariate Correlations*

|  | | **Frailty Dimensions** | | | | | | | | | | | |  | **Sleep** | | |
| --- | --- | --- | --- | --- | --- | --- | --- | --- | --- | --- | --- | --- | --- | --- | --- | --- | --- |
|  |  | 1 | 2 | 3 | 4 | 5 | 6 | 7 | 8 | 9 | 10 | 11 | 12 |  | 1 | 2 | 3 |
| **Demographics** | 1. Age | .12^**^ | .17^***^ | .20^***^ | -.08^***^ | -.05^**^ | -.05 | -.06 | -.09 | -.07 | -.05 | .04 | .05 |  | .04 | .01 | -.02 |
|  | 2. Male | .05 | .09^***^ | .08^***^ | -.10^***^ | -.14^***^ | -.15 | -.01 | .02 | -.02 | .06 | -.03 | -.04 |  | .02 | .00 | .01 |
|  | 3. Employ.1 | -.01 | -.05^*^ | -.05^*^ | .02 | -.01 | -.02 | .02 | .02 | .01 | .08 | .02 | .01 |  | .00 | .01 | .01 |
|  | 4. Employ.2 | -.03 | -.03 | -.04 | -.03 | -.03 | -.02 | .07 | -.01 | -.01 | .08 | .05 | .03 |  | .01 | .02 | .02 |
|  | 5. Employ.3 | -.02 | -.04^*^ | -.02 | -.03 | -.04^*^ | -.03 | .07 | .02 | -.02 | .02 | .04 | .02 |  | .03 | .04 | .03 |
|  | 6. Alcohol.1 | -.08 | -.02 | .03 | -.01 | .00 | .00 | -.03 | .06 | .05 | -.02 | -.01 | -.01 |  | .00 | .01 | .01 |
|  | 7. Alcohol.2 | -.02 | .00 | .03 | -.03 | -.02 | -.02 | -.03 | .06 | .03 | -.04 | -.03 | -.03 |  | .02 | .01 | .03 |
|  | 8. Alcohol.3 | -.01 | .00 | .01 | -.04^*^ | -.04^*^ | -.03 | .02 | .06 | .03 | -.06 | -.04 | -.02 |  | .00 | .01 | .01 |
|  | 9. Smoke.1 | .06 | .06^**^ | .06^**^ | .03 | .01 | .03 | -.10 | .04 | -.02 | .04 | .07 | .05 |  | -.06 | -.08 | -.08 |
|  | 10. Smoke.2 | .08 | .04 | .02 | -.01 | -.03 | -.02 | .00 | .02 | .00 | .05 | .06 | .05 |  | -.04 | -.05 | -.07 |
|  | 11. Smoke.3 | -.01 | .02 | .02 | -.01 | -.02 | -.02 | -.02 | .01 | .01 | .05 | .04 | .02 |  | -.03 | -.02 | -.03 |
| **Frailty Dimensions** | 1. Physical.1 | - | .65^***^ | .54^***^ | .22^***^ | .16^***^ | .20^***^ | .18 | .06 | .02 | .18^***^ | .11^*^ | .11^*^ |  | -.17^***^ | -.19^***^ | -.22^***^ |
|  | 2. Physical.2 |  | - | .71^***^ | .20^***^ | .22^***^ | .18^***^ | -.01 | -.05^*^ | -.03 | .10^**^ | .14^***^ | .13^***^ |  | -.14^***^ | -.21^***^ | -.20^***^ |
|  | 3. Physical.3 |  |  | - | .20^***^ | .20^***^ | .20^***^ | -.02 | -.05^*^ | -.03 | .07 | .15^***^ | .17^***^ |  | -.15^***^ | -.20^***^ | -.19^***^ |
|  | 4. Psychological.1 |  |  |  | - | .70^***^ | .67^***^ | -.06 | -.02 | -.02 | .24^***^ | .25^***^ | .22^***^ |  | -.18^***^ | -.15^***^ | -.14^***^ |
|  | 5. Psychological.2 |  |  |  |  | - | .76^***^ | -.04 | -.02 | -.05^*^ | .16^***^ | .24^***^ | .22^***^ |  | -.15^***^ | -.18^***^ | .15^***^ |
|  | 6. Psychological.3 |  |  |  |  |  | - | -.04 | -.01 | -.01 | .17^***^ | .23^***^ | .26^***^ |  | -.14^***^ | -.14^***^ | -.16^***^ |
|  | 7. Cognitive.1 |  |  |  |  |  |  | - | .55^***^ | .50^***^ | .05 | -.02 | -.03 |  | .01 | .01 | -.05 |
|  | 8. Cognitive.2 |  |  |  |  |  |  |  | - | .54^***^ | .00 | -.03 | -.03 |  | .01 | -.02 | -.01 |
|  | 9. Cognitive.3 |  |  |  |  |  |  |  |  | - | .04 | -.02 | -.01 |  | -.02 | .00 | .00 |
|  | 10. Social.1 |  |  |  |  |  |  |  |  |  | - | .65^***^ | .59^***^ |  | -.08^*^ | -.11^***^ | -.11^***^ |
|  | 11. Social.2 |  |  |  |  |  |  |  |  |  |  | - | .71^***^ |  | -.12^***^ | -.15^***^ | -.13^***^ |
|  | 12. Social.3 |  |  |  |  |  |  |  |  |  |  |  | - |  | -.11^***^ | -.13^***^ | -.15^***^ |
| **Sleep** | 1. Sleep.1 |  |  |  |  |  |  |  |  |  |  |  |  |  | - | .55^***^ | .50^***^ |
|  | 2. Sleep.2 |  |  |  |  |  |  |  |  |  |  |  |  |  |  | - | .61^***^ |
|  | 3. Sleep.3 |  |  |  |  |  |  |  |  |  |  |  |  |  |  |  | - |

*Descriptive Statistics of Main Study Variables*

|  | **Minimum** | **1^st^ Quartile** | **M (SD)** | **3^rd^ Quartile** | **Maximum** |
| --- | --- | --- | --- | --- | --- |
| **Physical Frailty (0-11)** |  |  |  |  |  |
| Time 1 | 0.00 | 1.33 | 2.67 (1.35) | 3.42 | 8.00 |
| Time 2 | 0.00 | 2.00 | 3.05 (1.51) | 4.00 | 10.00 |
| Time 3 | 0.00 | 2.33 | 3.39 (1.59) | 4.33 | 9.00 |
|  |  |  |  |  |  |
| **Psychological Frailty (0-7)** |  |  |  |  |  |
| Time 1 | 0.00 | 0.00 | 1.30 (1.34) | 2.00 | 7.00 |
| Time 2 | 0.00 | 0.00 | 1.06 (1.23) | 2.00 | 7.00 |
| Time 3 | 0.00 | 0.00 | 1.07 (1.25) | 2.00 | 7.00 |
|  |  |  |  |  |  |
| **Cognitive Frailty (z-scores)** |  |  |  |  |  |
| Time 1 | -1.39 | -0.07 | 0.23 (0.48) | 0.53 | 2.25 |
| Time 2 | -2.27 | -0.26 | 0.05 (0.51) | 0.39 | 2.70 |
| Time 3 | -2.05 | -0.31 | 0.001 (0.50) | 0.33 | 1.59 |
|  |  |  |  |  |  |
| **Social Frailty (0-7)** |  |  |  |  |  |
| Time 1 | 0.60 | 1.50 | 2.06 (0.87) | 2.60 | 6.00 |
| Time 2 | 0.20 | 1.40 | 2.08 (0.89) | 2.60 | 5.60 |
| Time 3 | 0.40 | 1.40 | 2.17 (0.92) | 2.60 | 6.00 |
|  |  |  |  |  |  |
| **Sleep Quality (0-6)** |  |  |  |  |  |
| Time 1 | 0.00 | 3.00 | 3.96 (1.08) | 5.00 | 6.00 |
| Time 2 | 0.00 | 3.00 | 3.99 (1.06) | 5.00 | 6.00 |
| Time 3 | 0.00 | 3.00 | 5.00 (1.05) | 5.00 | 6.00 |

Table 1 displays the distribution of each main variable at each time point. These data highlight that the most of participants scored in the lower end of the distribution for each frailty domain; that is at least 75% of participants showed low levels of frailty across each wave of assessment. Similarly, for sleep while around 25% of participants reported low sleep quality, most participants appear towards the upper end of the range, suggesting good sleep quality.

**Supplementary Table 5**

*RI-CLPM Covariate Statistics*

|  | **Physical Frailty**  **and Sleep** | | |  | **Psychological Frailty**  **and Sleep** | | |  | **Cognitive Frailty**  **and Sleep** | | |  | **Social Frailty**  **and Sleep** | | |
| --- | --- | --- | --- | --- | --- | --- | --- | --- | --- | --- | --- | --- | --- | --- | --- |
|  | ***β*** | ***SE*** | ***p*** |  | ***β*** | ***SE*** | ***p*** |  | ***β*** | ***SE*** | ***p*** |  | ***β*** | ***SE*** | ***p*** |
| **Time 2 – Frailty** |  |  |  |  |  |  |  |  |  |  |  |  |  |  |  |
| Age | **.001** | **.001** | **< .001** |  | **-.004** | **.00** | **< .001** |  | **-.01** | **.00** | **< .001** |  | **.002** | **.00** | **.009** |
| Male Gender | **.19** | **.06** | **< .001** |  | **-.25** | **.03** | **< .001** |  | .06 | .02 | .234 |  | **-.14** | **.03** | **.002** |
| T1 Employed | **-.14** | **.05** | **< .001** |  | -.02 | .03 | .648 |  | .02 | .02 | .676 |  | -.004 | .03 | .924 |
| T1 Daily Alcohol | -.09 | .06 | .069 |  | .01 | .03 | .846 |  | **.17** | **.02** | **.004** |  | -.01 | .03 | .838 |
| T1 Smoker | .18 | .13 | .075 |  | **-.21** | **.07** | **.042** |  | **.32** | **.04** | **.007** |  | **.29** | **.06** | **.005** |
|  |  |  |  |  |  |  |  |  |  |  |  |  |  |  |  |
| **Time 2 – Sleep** |  |  |  |  |  |  |  |  |  |  |  |  |  |  |  |
| Age | .001 | .00 | .199 |  | .001 | .00 | .156 |  | .001 | .00 | .221 |  | .001 | .00 | .248 |
| Male Gender | -.04 | .03 | .380 |  | -.06 | .03 | .189 |  | -.04 | .03 | .304 |  | -.04 | .03 | .367 |
| T1 Employed | .03 | .03 | .460 |  | .03 | .03 | .448 |  | .03 | .03 | .429 |  | .04 | .03 | .344 |
| T1 Daily Alcohol | .05 | .03 | .317 |  | .05 | .03 | .268 |  | .06 | .03 | .235 |  | .05 | .03 | .325 |
| T1 Smoker | **-.24** | **.07** | **.014** |  | **-.24** | **.07** | **.014** |  | **-.24** | **.07** | **.012** |  | **-.24** | **.07** | **.012** |
|  |  |  |  |  |  |  |  |  |  |  |  |  |  |  |  |
| **Time 3 – Frailty** |  |  |  |  |  |  |  |  |  |  |  |  |  |  |  |
| Age | **.01** | **.001** | **< .001** |  | **-.003** | **.00** | **< .001** |  | **-.01** | **.00** | **< .001** |  | **.004** | **.00** | **< .001** |
| Male Gender | .04 | .05 | .333 |  | **-.31** | **.03** | **< .001** |  | -.03 | .02 | .657 |  | **-.15** | **.03** | **< .001** |
| T2 Employed | -.02 | .07 | .714 |  | .06 | .04 | .326 |  | .06 | .03 | .410 |  | -.001 | .04 | .989 |
| T2 Daily Alcohol | .07 | .06 | .159 |  | .02 | .04 | .678 |  | .08 | .03 | .274 |  | -.001 | .03 | .981 |
| T2 Smoker | .03 | .15 | .794 |  | -.10 | .09 | .467 |  | .10 | .07 | .565 |  | .19 | .08 | .134 |
|  |  |  |  |  |  |  |  |  |  |  |  |  |  |  |  |
| **Time 3 – Sleep** |  |  |  |  |  |  |  |  |  |  |  |  |  |  |  |
| Age | .001 | .00 | .255 |  | .001 | .00 | .211 |  | .001 | .001 | .080 |  | .001 | .01 | .154 |
| Male Gender | .01 | .03 | .787 |  | -.01 | .03 | .836 |  | -.001 | .001 | .974 |  | -.01 | .03 | .837 |
| T2 Employed | .05 | .04 | .466 |  | .05 | .04 | .402 |  | .06 | .05 | .361 |  | .06 | .04 | .299 |
| T2 Daily Alcohol | .08 | .04 | .122 |  | .09 | .04 | .111 |  | .08 | .04 | .146 |  | .09 | .04 | .130 |
| T2 Smoker | **-.44** | **.10** | **.001** |  | **-.46** | **.10** | **.001** |  | **-.47** | **.10** | **< .001** |  | **-.41** | **.10** | **.002** |


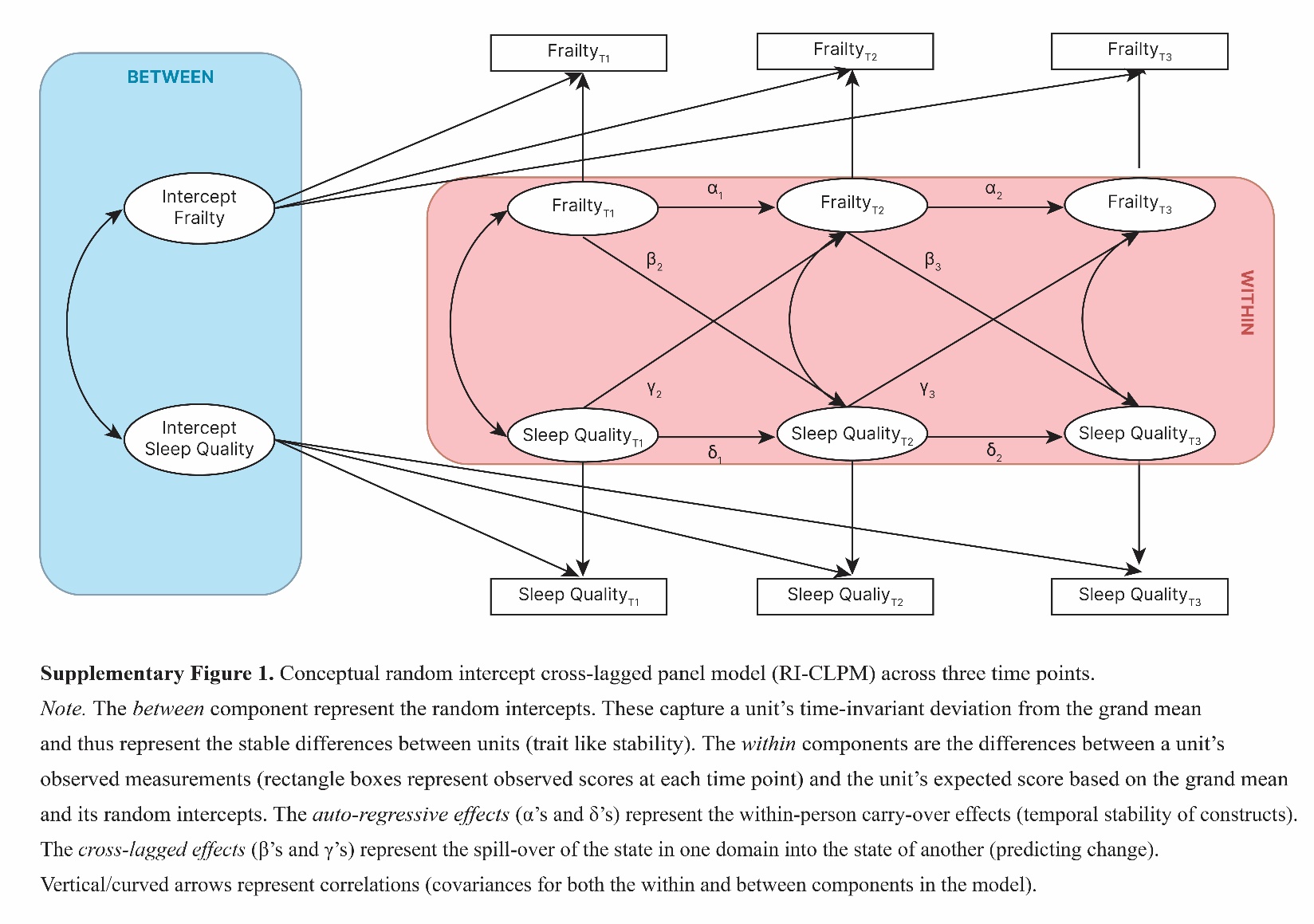
**Supplementary 6**

***Supplementary Figure 1.*** Conceptual random intercept cross-lagged panel model (RI-CLPM) across three time points.

*Note.* The *between* component represent the random intercepts. These capture a unit’s time-invariant deviation from the grand mean and thus represent the stable differences between units (trait like stability). The *within* components are the differences between a unit’s observed measurements (rectangle boxes represent observed scores at each time point) and the unit’s expected score based on the grand mean and its random intercepts. The *auto-regressive effects* (α’s and δ’s) represent the within-person carry-over effects (temporal stability of constructs). The *cross-lagged effects* (β’s and γ’s) represent the spill-over of the state in one domain into the state of another (predicting change). Vertical/curved arrows represent correlations (covariances for both the within and between components in the model).
